# Proteomic analysis of human cervical adenocarcinoma mucus to identify potential protein biomarkers

**DOI:** 10.7717/peerj.9527

**Published:** 2020-07-28

**Authors:** Zhifang Ma, Jie Chen, Ting Luan, Chengzhuo Chu, Wangfei Wu, Yichao Zhu, Yun Gu

**Affiliations:** 1 Department of Pathology, Women’s Hospital of Nanjing Medical University, Nanjing Maternity and Child Health Care Hospital, Nanjing, China; 2 Department of Gynecology, Women’s Hospital of Nanjing Medical University, Nanjing Maternity and Child Health Care Hospital, Nanjing, China; 3 Department of Physiology, Nanjing Medical University, Nanjing, China; 4 State Key Laboratory of Reproductive Medicine, Nanjing Medical University, Nanjing, China

**Keywords:** Cervical adenocarcinoma, Mucus, Proteomics, Biomarkers

## Abstract

**Background:**

Cervical cancer is the most common gynecological cancer, encompassing cervical squamous cell carcinoma, adenocarcinoma, and other epithelial tumors. There are many diagnostic methods to detect cervical cancers but no precision screening tool for cervical adenocarcinoma at present.

**Material and methods:**

The cervical mucus from three normal cervices (Ctrl), three endocervical adenocarcinoma (EA), and three cervical adenocarcinoma in situ (AIS) was collected for proteomic analysis. The proteins were screened using liquid chromatography-mass spectrometry analysis (LC-MS). The biological function of the differently expressed proteins were predicted by Gene Ontology (GO).

**Results:**

A total of 711 proteins were identified, including 237 differently expressed proteins identified in EA/Ctrl comparison, 256 differently expressed proteins identified in AIS/Ctrl comparison, and 242 differently expressed proteins identified in AIS/EA comparison (up-regulate ≥ 1.5 or down-regulate ≤ 0.67). Functional annotation was performed using GO analysis on 1,056 differently expressed proteins to identify those that may impact cervical cancer, such as heme protein myeloperoxidase, which is involved in the immune process, and APOA1, which is associated with lipid metabolism.

**Conclusion:**

We used proteomic analysis to screen out differently expressed proteins from normal cervical mucus and cervical adenocarcinoma mucus samples. These differently expressed proteins may be potential biomarkers for the diagnosis and treatment of cervical adenocarcinoma but require additional study.

## Introduction

Cervical cancer is the third most commonly diagnosed gynecological malignancy and the fourth leading cause of cancer-related death in women worldwide resulting in approximately 530,000 deaths annually (Siegel et al., 2014; Bray et al., 2018). Advances in the treatment of cervical cancer, such as radiotherapy, chemotherapy, surgery, immunotherapy, and targeted therapy have not improved the survival rate for cervical cancer (Dai et al., 2016; Menderes et al., 2016). Cervical adenocarcinoma follows squamous cell carcinoma as the most common subtype of cervical carcinoma and is increasing in incidence and prevalence in younger populations (Seoud, Tjalma & Ronsse, 2011). Novel biomarkers for the diagnosis and treatment of cervical adenocarcinoma are needed.

A number of studies have looked at the prevention and treatment of cervical cancer by investigating relevant cells, tissues, and subtypes (Farzanehpour et al., 2019; Li, Hong & Wijayakulathilaka, 2019; Siegler et al., 2019). Cervical cancer is commonly diagnosed through cytological screening known as a Pap test in conjunction with the detection of high-risk human papillomaviruses (hr-HPVs). However, these tests are expensive and depend on good infrastructure and well-trained personnel (Lynge et al., 2014). Proteomics analysis is a powerful tool for monitoring the change of protein levels to discover new biomarkers in many cancers, such as colorectal cancer, pancreatic cancer, and neuroendocrine cervical cancer (Lin et al., 2014; Pan, Brentnall & Chen, 2015; Chauvin & Boisvert, 2018). Unique membrane proteins have been identified using proteomics analysis of the cervical cancer cell lines (Pappa et al., 2018). Lee et al. (2011) studied the proteome of cervical mucus plugs and suggested its role for maintaining pregnancy and parturition. However, their approach was not suitable for comparative studies of mucins among different groups. The constitutive protein composition of cervical mucus in fertile women and changes in the cervical mucus proteome were identified throughout the menstrual cycle but interactions between immunoglobulin, defense-binding protein, and other cervical mucus proteins were not studied (Grande et al., 2015).

We sought to discover novel biomarkers using proteomics analysis of nine uterine cavity mucus samples to produce a better prognosis for cervical cancer and to provide a reliable reference for the diagnosis and treatment of patients with cervical adenocarcinoma.

## Materials and Methods

### Patient materials

Cervical mucus samples were obtained from three patients with normal cervices (Ctrl), three patients with endocervical adenocarcinoma (EA), and three patients with adenocarcinoma in situ of the cervix (AIS) who had a total hysterectomy at the Nanjing Maternity and Child Health Care Hospital Affiliated to Nanjing Medical University. The three normal cervical mucus samples acted as the control. All samples were stored at −80 °C and an initial histopathological diagnosis was conducted. The cancerous cervical mucus and normal cervical mucus were used for proteomic analyses. This study was approved by the ethical committee of Women’s Hospital of Nanjing Medical University, Nanjing Maternity and Child Health Care Hospital ((2019) KY-040). All participants voluntarily agreed to participate in this study and signed an informed consent.

### Protein extraction

Protein extraction was performed on the nine cervical mucus samples. Each sample was mixed and homogenized with a protein lysate (7 M Urea, 2 M Thiourea, 4% SDS, 40 mM Tris–HCl, pH 8.5, 1 mM Phenylmethanesulfonyl fluoride (PMSF), 2 mM Ethylene Diamine Tetraacetic Acid (EDTA)) on ice for 5 min. Dithiothreitol (DTT) (Solarbio, 428F0422) was added to a final concentration of 10 mM, followed by an ice bath ultrasound for 15 min and centrifuging at 13,000*g* at 4 °C for 20 min. After ultracentrifugation, supernatants of each samples were collected to a new centrifugal tube. Cold acetone was added into the centrifugal tube at four times the volume and was left to stand overnight at −20 °C. The protein precipitation was collected and left to air dry. A total of 8 M urea/100 mM tetraethyl-ammonium bromide (TEAB) (BCBC6216; Sigma–Aldrich, St. Louis, MO, USA) (pH 8.0) solution was added to re-dissolve the protein and then DTT was added to the final concentration of 10 mM and the solution was immersed in water at 56 °C for 30 min for the reduction reaction. Iodoacetamide (IAM, Aladdin, J1513091) was added to the final concentration of 55 mM and placed at room temperature in the dark for 30 min to produce an alkylation reaction. The Bradford method was used to measure protein concentrations.

### iTRAQ labeling and peptide fractionation

The extracted proteins were digested by trypsin to obtain the corresponding peptides, which were desalted by the Durashell C18 column (5 ms, 100 A, 4.6 × 250 mm) (Agela, Tianjin, China) and vacuum dried. The peptides were dissolved with 0.5 M TEAB and labeled using the itraq-8 standard kit (SCIEX, Shanghai, China) according to the manufacturer’s instructions. The samples were labeled and mixed and the mixed peptides were then graded and separated using the Ultimate 3000 HPLC system (DINOEX; Thermo, Waltham, MA, USA). The separation of peptides was achieved by increasing acetonitrile (CAN) concentration under alkaline conditions. The flow rate was 1 ml/min with one tube collected per minute. A total of 42 secondary fractions were collected and combined into 12 fractions, which were desalinated and vacuum-dried on the Strata-X column. All analyses were completed by Wuhan Genecreate Biological Engineering Co. LTD. (Wuhan, China).

### Protein identification and quantification

Proteins were identified by the Proteinpilot™ V4.5 search engine. At least one unique peptide section of each protein line and the unused score was believed to be no more than 1.3 (just above 95%). The reliability of each identified peptide and its protein quantification was considered for protein quantification.

### Liquid chromatography-mass spectrometry analysis

Mass spectrometry data were collected using the TripleTOF 5600+ liquid/mass coupling system (SCIEX, Shanghai, China). Polypeptide samples were dissolved in 2% acetonitrile/0.1% formic acid and analyzed using the TripleTOF 5600+ mass spectrometer. The peptide solution was added to the C18 capture column (5 ms, 100 ms × 20 mm) and eluted on the C18 analysis column (3 ms, 75 ms × 150 mm) for 90 min with a flow rate of 300 nL/min. The two mobile phases were buffer A (2% acetonitrile, 0.1% formic acid, 98% H_2_O) and buffer B (98% acetonitrile, 0.1% formic acid, 2% H_2_O). The primary mass spectrometry for information dependent acquisition (IDA) was scanned at 250 ms ion accumulation time and a secondary mass spectrometry of 30 precursor ions was collected at 50 ms ion accumulation time. The MS1 spectrum was collected in the range of 350–1,500 m/z, and the MS2 spectrum was collected in the range of 100–1,500 m/z. The dynamic exclusion time for precursor ions was set at 15 s.

### Functional annotation

Gene ontology (GO) functional annotation analysis was performed for all identified proteins and the GO functions of the cellular component, biological process, and molecular function corresponding to all proteins were analyzed. Detailed information can be found at http://www.geneontology.org. COG analysis and KEGG analysis were used to further determine the function of the proteins.

### Statistics

The mean value of the ratio of repeated samples was normalized using the median as the difference multiple by the samples to be compared. The minimum *P* value of Student’s *t* test of the single sample of parewise comparisons between repeated samples was used as the significance difference of the samples. Differential proteins were screened according to fold change and *P* value. The proteins were considered to be statistically different when the difference of multiples was ≥1.5 (i.e., up-regulate ≥1.5 and down-regulate ≤0.67) with a *P* value < 0.05 after the significance statistical test.

## Results

### Identification of proteins in cervical mucinous

**Figure 1:**
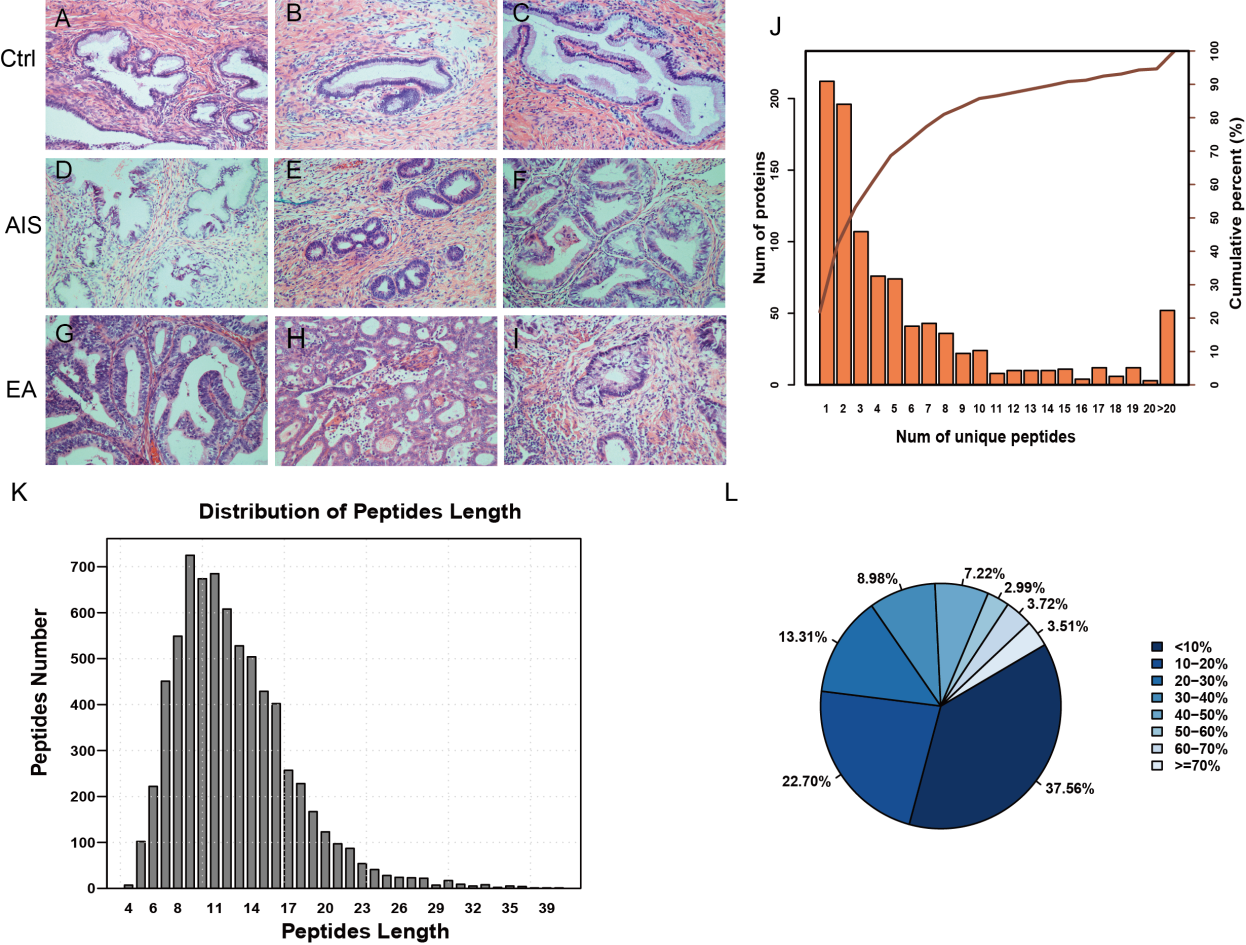
Identification of proteins related to cervical mucus. (A–C), pathological section analysis of normal cervical mucus (HE, ×200); (D–F), pathological section analysis of cervical adenocarcinoma mucus in situ (HE, ×200); (G–I), pathological section analysis of endocervical adenocarcinoma mucus (HE, ×200). (J) All peptide length profiles. (K) The distribution of unique peptide number of identified proteins. (L) Pie chart of protein identification coverage distribution.

A total of nine samples were divided into three groups to identify differently abundant proteins. Ctrl was comprised of samples from three healthy individuals, EA was comprised of samples from three invasive cervical adenocarcinoma individuals, and AIS had samples from three cervical adenocarcinoma in situ individuals. All samples were obtained from individuals with an average age of 41 years. The clinicopathological characteristics of these patients were summarized in Table 1. A diagnosis of endocervical adenocarcinoma was suggested by the cervical sample based on cytomorphological features. The postoperative pathological diagnosis showed no lymph node metastasis and immunohistochemical results directed the diagnosis and subsequent treatment. The corresponding pathological images were obtained for the nine samples and were stained by HE (Fig. 1A; Fig. S1).

**Table 1:** Patient information of samples used in the comprehensive proteomic experiments.

Sample	Age (years old)	FIGO stage	Lesion size (mm)	Depth of invasion	Immunohistochemical diagnosis	Chief complaint	TCT	HPV
Ctrl-1	49	Normal				Routine physical	−	−
Ctrl-2	44	Normal				Routine physical	−	−
Ctrl-3	40	Normal				Routine physical	−	−
AIS-1	36	0	2.3		Ki67(70%+), P16(+), ER(−)	Cervical lesions	ASC-H	+
AIS-2	32	0	2		Ki67(90%+), P16(+), ER(−)	Vaginal bleeding after intercourse	+	+
AIS-3	43	0	<7		Ki67(50%+), P16(+), ER(+)	Cervical lesions	−	+
EA-1	54	II A1	12	>1/3 & <2/3	Ki67(60%+), P16(+), ER(−)	Vaginal bleeding after menopause	NK	NK
EA-2	37	I B2	≤20	>1/3 & <2/3	Ki67(2%+), P16(++), ER(+)	Menstrual disorder and abnormal leukorrhea	ASC-US	+
EA-3	34	II A1	30	>1/3 & <2/3	Ki67(80%+), P16(−), ER(−)	Vaginal bleeding after intercourse	HSIL	+

**Note:**

Ctrl indicates the normal cervix; AIS indicates adenocarcinoms in situ of the cervical; EA indicates endocervical adenocarcinoma; TCT, Thinprep cytology test; HPV, human papillomavirus; −, negative; +, positive; NK, not known; ASC-H, atypical squamous cells: cannot exclude high-grade squamous intraepithelial lesion; ASC-US, atypical squamous cells of undetermined significance; HSIL, high-grade squamous intraepithelial lesions.

The unique peptides only needed to be detected in one protein to identify the protein in the sample. The presence of the peptide uniquely determined the presence of the corresponding protein. The biordinate distribution of the unique peptides representing the unique proteins was identified (Fig. 1B). The abscissa is the number of unique peptides contained in the protein, and the left ordinate is the number of proteins corresponding to the abscissa. The right ordinate is the cumulative protein proportion corresponding to the abscissa. The results showed that there are 757 protein with at least 2 unique peptides, accounting for 78.12% of the total protein. The length distribution of the peptide was obtained by mass spectrometer (Fig. 1C). The average length of the peptides identified was 12.55, which was a reasonable peptide length. A protein with more peptide support makes the protein more reliable, therefore, protein coverage can indirectly reflect the overall accuracy of the identification results. Pie charts were used to represent the percentage of different proteins within the identification coverage (Fig. 1D). The identified range of proteins was 37.56% and proteins with coverage ≥20% accounted for 39.73% of the total protein. The average protein coverage was 21.90%.

### The analysis of differential protein expression profile

**Figure 2:**
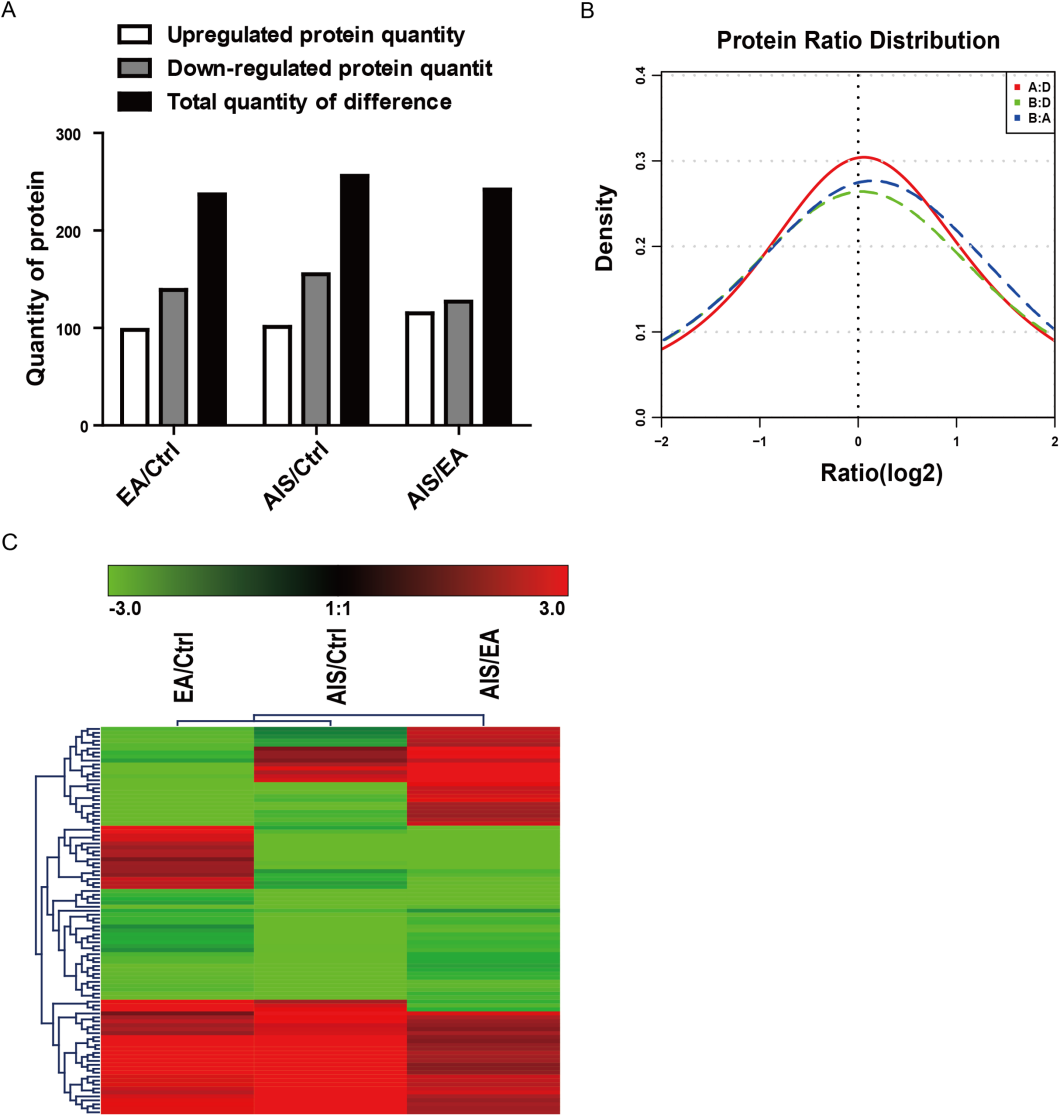
Distribution and stratification of differential proteins. (A) Distribution of differential proteins between two groups. (B) Protein abundance distribution map. The figure shows the distribution of the differential multiples of all quantifiable proteins, where the *x*-coordinate represents the value of the differential multiples after logarithmic transformation in base 2. The part greater than 0 indicates the up-regulated expression, while the part less than 0 indicates the down-regulated expression. (C) Hierarchical clustering heat map of differential protein layers. Rows represent protein clustering and the column represents the sample pair clustering situation. As the protein ratio changes from small to large, the heat map color shows a corresponding green-black-red change.

Of the identified proteins, 237, 256, and 242 proteins were significantly differently expressed in the comparison group EA/Ctrl, AIS/Ctrl, and AIS/EA using iTRAQ quantitative analysis with the filtered threshold of up-regulation ≥1.5 or down-regulation ≤0.67, respectively. The number of significant protein differences was shown between two samples (Fig. 2A). The results of the relative quantitative analysis of the protein showed that the protein ratio distribution followed a normal distribution pattern. The distribution of the differential multiples of all quantifiable proteins was shown (Fig. 2B), where the *x*-coordinate represented the value of the differential multiples after logarithmic transformation in base 2. The part greater than 0 indicated the up-regulated expression, while the part less than 0 indicated the down-regulated expression. The hierarchical clustering heat map of the intersection of 98 differently significant proteins in the comparison groups visually aggregated samples/proteins with higher similarity (Fig. 2C). The data source is the log2 logarithmic value of the protein abundance ratio between the two comparison groups. Quantitative information of some significantly different proteins is shown in Table 2. Some of the differently expressed proteins were significantly expressed in the groups EA/Ctrl, AIS/Ctrl, and AIS/EA, including myeloperoxidase and APOA1, which are involved in the development of cancer (Cine et al., 2014; Castelao et al., 2015; Zadeh Fakhar et al., 2019).

**Table 2:** Quantitative information on the top 10 proteins with significant differences among different components.

	Accession	Gene	Protein.names	*P*-value
Up-regulated				
EA:Ctrl	P04114	APOB	Apolipoprotein	0.0402
	P02751	FINC	Fibronectin	0.0069
	P13646	K1C13	Keratin, type I cytoskeletal 13	0.001
	P02549	SPTA1	Spectrin alpha chain, erythrocytic 1	0.0003
	P04040	CATA	Catalase	0.0034
	P19013	K2C4	Keratin, type II cytoskeletal 4	0.0006
	P05164	PERM	Myeloperoxidase	0.0007
	P11277	SPTB1	Spectrin beta chain, erythrocytic	0.0024
	P13796	PLSL	Plastin-2	0.0082
	P01833	PIGR	Polymeric immunoglobulin receptor	0.0003
AIS:EA	P67775	PP2AA	Serine/threonine-protein phosphatase 2A catalytic subunit alpha isoform	0.000000115
	P69892	HBG2	Hemoglobin subunit gamma-2	0.00000523
	Q13228	SBP1	Methanethiol oxidase	0.0001
	P02656	APOC3	Apolipoprotein C-III	0.0001
	P07476	INVO	Involucrin	0.0003
	P01019	ANGT	Angiotensinogen	0.0004
	P68371	TBB4B	Tubulin beta-4B chain	0.0005
	P07195	LDHB	L-lactate dehydrogenase B chain	0.0006
	P00352	AL1A1	Retinal dehydrogenase 1	0.0008
	P02647	APOA1	Apolipoprotein A–I	0.0009
AIS:Ctrl	Q8NFI4	F10A5	Putative protein FAM10A5	0.0000000278
	P63208	SKP1	S-phase kinase-associated protein 1	0.000000389
	P69892	HBG2	Hemoglobin subunit gamma-2	0.0001
	Q6XQN6	PNCB	Nicotinate phosphoribosyltransferase	0.0001
	P00918	CAH2	Carbonic anhydrase 2	0.0002
	P00915	CAH1	Carbonic anhydrase 1	0.0005
	P27169	PON1	Serum paraoxonase/arylesterase 1	0.0005
	P02549	SPTA1	Spectrin alpha chain, erythrocytic 1	0.0006
	P30043	BLVRB	Flavin reductase (NADPH)	0.0006
	P04040	CATA	Catalase	0.0006
Down-regulated				
EA:Ctrl	P0C0L5	CO4B	Complement C4-B	0.0064
	P01009	A1AT	Alpha-1-antitrypsin	0.0000254
	P08603	CFAH	Complement factor H	0.0014
	A8K2U0	A2ML1	Alpha-2-macroglobulin-like protein 1	0.0025
	P02647	APOA1	Apolipoprotein A–I	0.0027
	P00738	HPT	Haptoglobin	0.0078
	P02675	FIBB	Fibrinogen beta chain	0.0108
	Q09666	AHNK	Neuroblast differentiation-associated protein AHNAK	0.0002
	P04083	LPC1	Calpactin II	0.0003
	P02671	FIBA	Fibrinogen alpha chain	0.0034
AIS:EA	P0DOX2	IGA2	Immunoglobulin alpha-2 heavy chain	0.0000343
	P04792	HSP27	Heat Shock 27 kDa Protein 1	0.0000452
	P05164	PERM	Myeloperoxidase	0.0001
	P02751	FINC	Fibronectin	0.0001
	P13647	K2C5	Keratin, type II cytoskeletal 5	0.0002
	P06702	MRP14	Migration inhibitory factor-related protein 14	0.0003
	P27797	CALR	Calreticulin	0.0003
	P25815	S100P	Protein S100-P	0.0004
	P07737	PROF1	Profilin-1	0.0007
	P15104	GLNA	Glutamine synthetase	0.0007
AIS:Ctrl	P14618	KPYM	Pyruvate kinase PKM	0.0000178
	P35321	SPR1A	Cornifin-A	0.0001
	P59665	MRS	Myeloid-Related Sequence	0.0002
	P05164	PERM	Myeloperoxidase	0.0003
	P02750	A2GL	Leucine-rich alpha-2-glycoprotein	0.0003
	P02748	CO9	Complement component C9	0.0004
	P31151	S10A7	Protein S100-A7	0.0005
	Q9UBG3	Sep-53	Squamous epithelial heat shock protein 53	0.0006
	P80188	NGAL	Neutrophil gelatinase-associated lipocalin	0.0006
	O60218	AK1BA	Aldo-keto reductase family 1 member B10	0.0007

### Functional and pathway analysis of identified proteins

**Figure 3:**
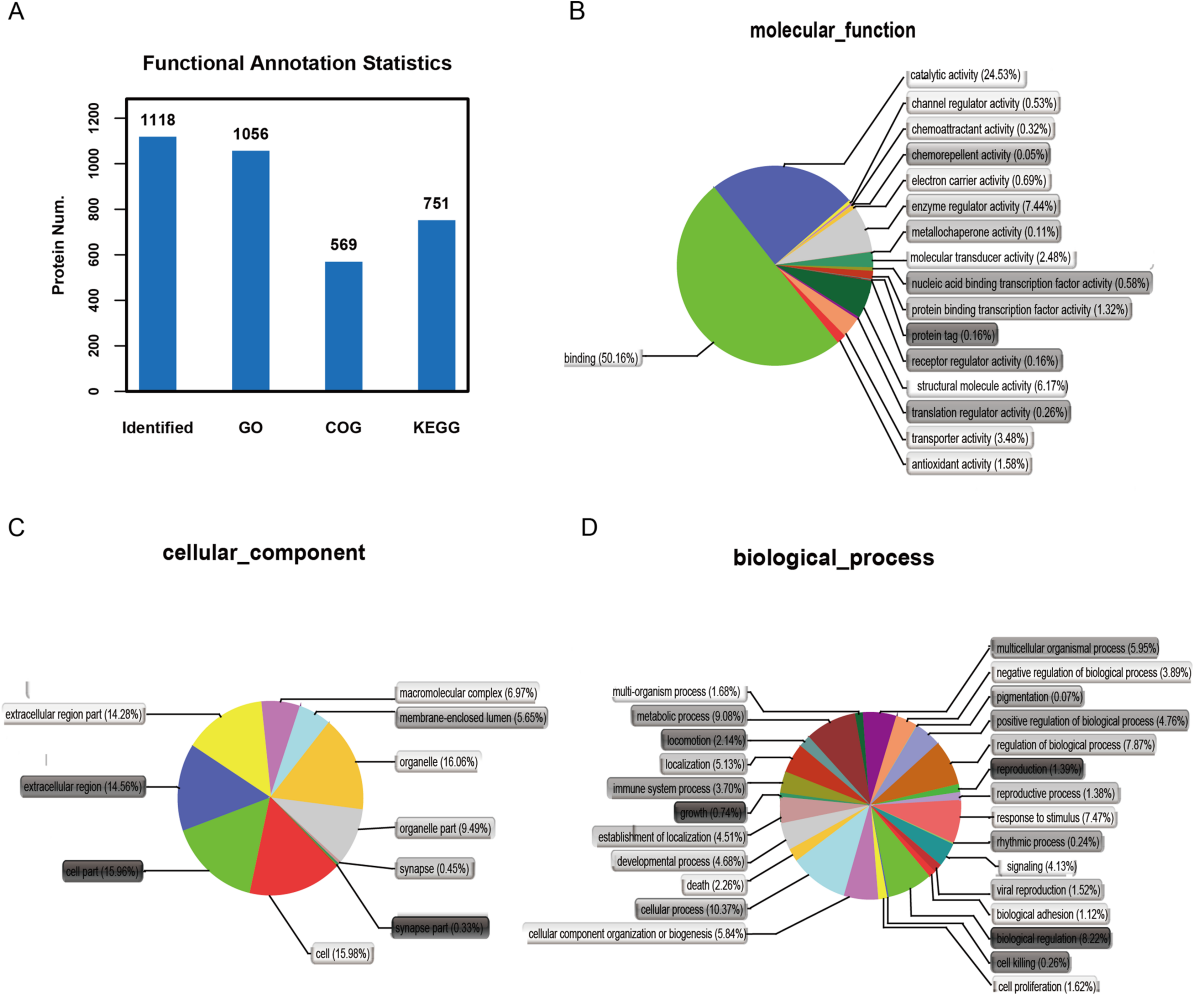
Functional annotation of differential proteins. (A) Different function annotation result statistics graph. The *x*-coordinate represents identification or different annotation methods, and the *y*-coordinate represents the number of proteins corresponding to the *x*-coordinate. Classification of 1,056 differently expressed proteins in three groups of cervical mucus samples according to (B) biological process, (C) cellular component and (D) molecular function by GO analysis. Statistical chart of functional annotation results of different proteins GO.

**Figure 4:**
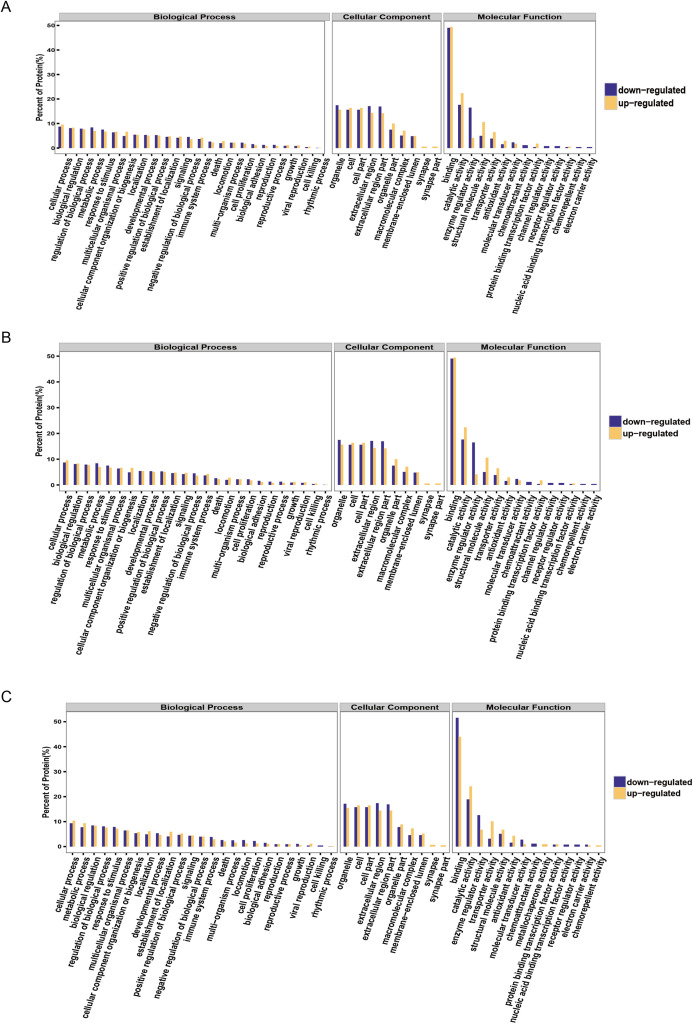
GO function annotation statistics results of differential proteins. The yellow column shows that (A) EA/Ctrl (B) AIS/EA (C) AIS/Ctrl upregulation of the functional classification proportion of differential proteins under three standard bodies. The green column is the functional classification proportion under the three ontologies of down-regulated differential proteins.

It is important to analyze the function of genes as more genomes are sequenced and to predict the function of unknown genes to guide further experiments. We provided the commonly used GO annotation and KEGG annotation results, as well as the COG annotation results to comprehensively reflect the function of proteins obtained from different databases to reveal the biological significance of proteins in various life activities. A total of 1,118 proteins were identified and functionally annotated (Fig. 3A). The annotation information for some proteins varied among the databases due to the limitations of the background annotation library. GO, COG, and KEGG annotated 1,056 proteins, 569 proteins, and 751 proteins, respectively. GO is a community-based bioinformatics resource that supplies information about gene product function using ontologies to represent biological knowledge (Gene Ontology Consortium, 2015). GO was used to annotate 1,056 proteins and statistical analysis was performed based on biological, cellular, and molecular processes (Figs. 3B–3D). We conducted independent functional annotation analysis on the up-regulated and down-regulated differently expressed proteins to better analyze the functions of differently expressed proteins. A total of 237 differently expressed proteins (98 significantly up-regulated and 139 significantly down-regulated proteins) in EA/Ctrl were used as examples to compare and analyze their GO annotation results. Functional notes were analyzed for groups AIS/Ctrl and AIS/EA, and the results are shown in a bar chart in Figs. 4A–4C.

## Discussion

Cervical cancer is an aggressive gynecological cancer of the uterine cervix. It is the fourth most common cancer among women worldwide, with an estimated 570,000 cases and 311,000 deaths in 2018 (Bray et al., 2018; Takayanagi et al., 2019). Although the incidence of cervical squamous cell carcinoma has decreased, the incidence of cervical adenocarcinoma remains high (Vizcaino et al., 1998; Smith et al., 2000; Fan et al., 2014; Saida et al., 2019; Ouyang et al., 2020). Cervical adenocarcinoma currently accounts for up to 25% of all cases of cervical cancer in many western countries (Parkin & Bray, 2006). It is important to find novel biomarkers relevant to cervical adenocarcinoma to improve the current treatment strategies and prognosis of this disease.

We identified differently expressed proteins from subjects with cervical adenocarcinoma and those with normal cervices using the iTRAQ proteomics approach. A total of 1,118 proteins were identified in three separate trials with 711 common proteins identified. We found that the number of proteins with significant differences in the comparison groups (EA/Ctrl, AIS/Ctrl and AIS/EA) was 237, 256 and 242, respectively. We performed functional annotations, including GO, COG, and pathway analysis, on significantly differently expressed proteins. Expression level clustering analysis and functional enrichment analysis was performed on all significantly differently expressed proteins to determine significant proteins.

There are many studies on the proteomics of cervical cancer but few studies on the proteomics analysis of cervical adenocarcinoma (Kacerovsky & Tosner, 2009; Serafín-Higuera et al., 2016; Zhang et al., 2016). We performed proteomics analysis on samples of cervical adenocarcinoma mucus and normal cervical mucus a nd used functional annotation to screen out differently expressed proteins relevant to immune, metabolic, cell adhesion, and other cellular processes, such as myeloperoxidase and APOA1. Differently expressed proteins may be used as markers for the early diagnosis of cervical adenocarcinoma, however, our study had some limitations, including its small sample size and lack of other techniques to verify the accuracy of our analysis.

Myeloperoxidase, also known as MPO or PERM, is an important member of the heme peroxidase-cyclooxygenase superfamily. It is mainly expressed in neutrophils and monocytes (Ndrepepa, 2019). The enzyme can be released through phagocytosis in phagocytic bodies to catalyze the synthesis of hypochlorous acid (HOCl) from hydrogen peroxide (H_2_O_2_) and chloride ions (Cl^−^) (Arnhold & Flemmig, 2010; Gomez-Mejiba et al., 2010; Rossmann et al., 2011). HOCl is a potent microbicidal agent that damages DNA, proteins, and lipids. Myeloperoxidase is associated with many cancer types, including lung, ovarian, colorectal, and prostate cancers (Arslan, Pinarbasi & Silig, 2011; Li et al., 2011; Castillo-Tong et al., 2014; Zou et al., 2018) through the −463G/A promoter polymorphism. It is reported that the −463G/A MPO gene polymorphism is not associated with cervical intraepithelial neoplasia and susceptibility to cervical cancer and the genotype GG of MPO with a higher transcriptional activity is a protective against cervical cancer (Mustea et al., 2007; Castelao et al., 2015; Natter et al., 2016). Additional studies are required to determine the role of myeloperoxidase in cervical tumorigenesis.

APOA1 belongs to the apolipoprotein A1/A4/E family and participates in lipid metabolism, including the intracellular reuse of fatty acids; it is an important carrier and cofactor (Zhang & Yang, 2018). APOA1 is the major component of high density lipoprotein. Rhee, Byrne & Sung (2017) reported that the ratio of HDL cholesterol to APOA1 may be a risk marker for cancer mortality. APOA1 is dysfunctional in cervical squamous cell carcinoma and is identified as a biomarker (Guo et al., 2015). We suggest that APOA1 may be a novel candidate marker for cervical adenocarcinoma and further study is needed to determine its functional mechanisms.

We collected nine cervical mucus samples and summarized their clinical information. Menopause is a life phenomenon with characteristics of estrogen secretion depletion and the cessation of menstruation. The majority of women enter menopause between the ages of 49 and 52 (Takahashi & Johnson, 2015) and some studies show no statistically significant difference in cervical cancer rates of premenopausal and postmenopausal women (Holt et al., 2017; Zhang et al., 2018). A total of 8 of the 9 individuals used in this study were premenopausal and the postmenopausal individual was within the age range of normal menopause. All samples met the guidelines for normal menstruation and had a uterus of normal size and position; the cervix was enlarged or inflamed in all nine cases. Our sample size was small due to variable sample collection time, long cycles, and limited scientific research funds. We intend to verify our results in the future and will investigate the mechanism of biomarkers used in the treatment of cervical adenocarcinoma.

## Conclusion

We performed a non-targeted proteomics study to profile differently expressed proteins in cervical adenocarcinoma. The proteins studied may serve as potential biomarkers for cervical cancer research and treatment.

## Supplemental Information

10.7717/peerj.9527/supp-1 Supplemental Information 1 The pathological section analysis of normal cervical mucus, of cervical adenocarcinoma mucus in situ, and of endocervical adenocarcinoma mucus (HE, ×200).

## Additional Information and Declarations

### Data Availability

The following information was supplied regarding data availability: The raw data are available in Figshare: Gu, Yun (2020): WIFF.zip. figshare. Dataset. DOI 10.6084/m9.figshare.11864337.v1.
