# Supplementary figures and images for: Proteomic analysis of human cervical adenocarcinoma mucus to identify potential protein biomarkers
Source: PeerJ. 2020 Jul 28;8:e9527. doi: 10.7717/peerj.9527 (PMC7394065; doi:10.7717/peerj.9527)

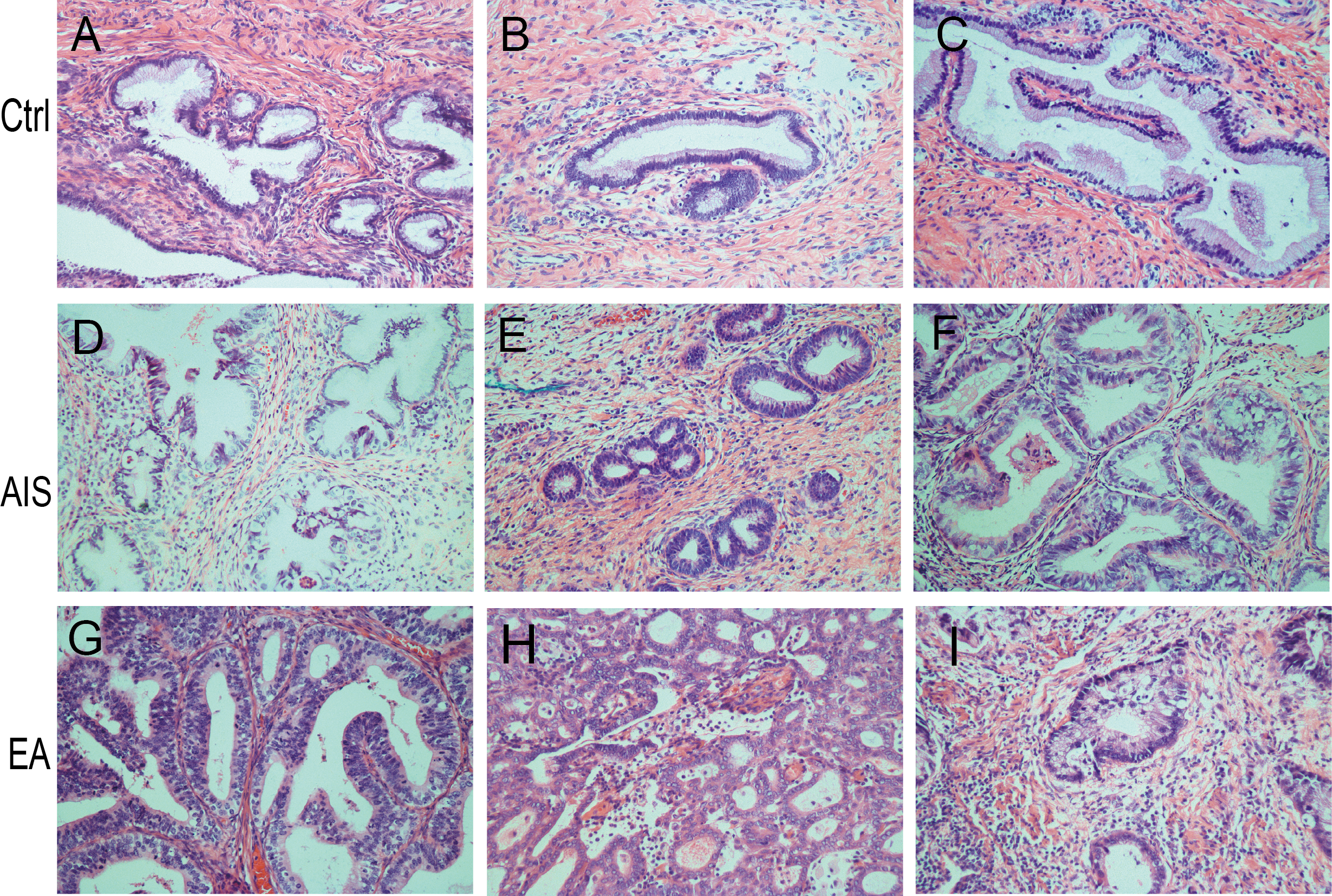

Supplement: Supplemental Information 1 [file peerj-08-9527-s001.png]
